# Improved Childhood Diarrhea Treatment Practices in Ghana: A Pre-Post Evaluation of a Comprehensive Private-Sector Program

**DOI:** 10.9745/GHSP-D-16-00021

**Published:** 2016-06-20

**Authors:** Marianne El-Khoury, Kathryn Banke, Phoebe Sloane

**Affiliations:** a Abt Associates, Inc., Bethesda, MD, USA

## Abstract

Diarrhea is the fourth leading cause of child mortality in Ghana. In 2010, Ghana endorsed guidelines from the World Health Organization and the United Nations Children’s Fund for use of zinc with low-osmolarity oral rehydration salts (ORS) for the treatment of acute childhood diarrhea. From late 2011 through 2014, the Strengthening Health Outcomes through the Private Sector (SHOPS) project implemented a comprehensive program in 3 regions of Ghana to increase the availability and use of ORS and zinc and to decrease incorrect use of antibiotics and antidiarrheals. The program included (1) partnering with local pharmaceutical firms to introduce and market locally produced zinc products, (2) collaborating with the Ghanaian Pharmacy Council to provide training and supportive supervision of private-sector providers on diarrhea management, and (3) conducting mass media campaigns to raise caregiver awareness. We evaluated the effect of this program using a baseline survey of 754 caregivers of children under 5 with diarrhea at the start of the intervention in 2012 and a follow-up survey of 751 caregivers in 2014. Regression analysis showed that use of ORS with zinc increased from 0.8% in 2012 to 29.2% in 2014 (*P*<.001), and antibiotic use declined from 66.2% to 38.2% (*P*<.001) during the same period. The magnitude and statistical significance of these results remained the same after including potential confounding factors as covariates. Inappropriate antibiotic use, however, remained high at follow-up. We conclude that similar programs applied in other settings have the potential to rapidly scale up use of ORS and zinc. Additional efforts are required to reduce persistent incorrect antibiotic use.

## Abstract (precis)

From 2011 to 2015, a diarrhea management program in Ghana targeting pharmaceutical suppliers, private-sector providers, and caregivers successfully increased caregiver use of oral rehydration salts (ORS) with zinc to treat diarrhea in children under 5, from 0.8% to 29.2%, and reduced antibiotic use (which is generally inappropriate for treatment of non-bloody diarrhea) from 66.2% to 38.2%.

## INTRODUCTION

Diarrhea is the third leading cause of death globally among children under the age of 5.^1^ Most of these deaths are related to dehydration and can easily be prevented with low-cost treatments such as oral rehydration salts (ORS).

In 2004, the World Health Organization (WHO) and the United Nations Children’s Fund (UNICEF) issued a joint statement endorsing the use of zinc together with a low-osmolarity formulation of ORS for the treatment of acute diarrhea (with no presence of blood in the stool or fever) among children.^2^ Numerous clinical trials demonstrated that, when given for 10 to 14 days during the course of an acute diarrhea episode, zinc reduces the duration and severity of the episode and prevents recurrence of diarrhea in the following 2 to 3 months.^3^^–^^6^ The benefits of zinc are substantial: it has been estimated that diarrhea mortality could be reduced by 23% with increased use of zinc to treat diarrhea.^7^ However, ORS and zinc remain underused, and antibiotics and/or antidiarrheals are often incorrectly administered instead.^8^^–^^10^

Zinc reduces the duration and severity of diarrhea episodes and has the potential to reduce diarrhea mortality by 23%.

Since 2005, donor-funded country programs have promoted the provision and use of ORS and zinc, while aiming to reduce incorrect treatment for children with diarrhea. For example, the United States Agency for International Development (USAID) has supported ORS and zinc projects in Benin, Ghana, India, Madagascar, Nepal, and Pakistan, among others. The Clinton Health Access Initiative (CHAI) has supported programs in India, Kenya, Nigeria, and Uganda. The Bill & Melinda Gates Foundation has supported a national zinc promotion program in Bangladesh and, along with UNICEF, the Children’s Investment Fund Foundation, and the Micronutrient Initiative, has also supported several pilot projects in India. Programmatic activities have generally included both public- and private-sector approaches focused on introducing high-quality and affordable zinc products to local markets in a sustainable way, using mass media campaigns and interpersonal communication to raise consumer knowledge of correct diarrhea treatment, and improving provider knowledge and skills through training and educational visits.

Despite the wealth of on-the-ground experience over the last decade, only a few studies have evaluated the effectiveness of such ORS and zinc interventions in improving correct management of acute childhood diarrhea. A recent evaluation in Bihar, India, found that children with diarrhea were almost 3 times as likely to be treated with ORS and zinc 2 years after an intervention designed to train public-sector providers and increase ORS and zinc availability.^11^ Another evaluation in India found that zinc use among children increased from 3% to 22% in Gujarat and from 3% to 7% in Uttar Pradesh, after training and supportive supervision interventions targeting public and private providers.^12^ In Bangladesh, over a 2-year period of implementing a national program to scale up zinc treatment, zinc use among children increased from less than 5% at baseline to 25%–30% in urban non-slum areas, 15%–20% in urban slums, and 9%–13% in rural areas.^13^ Six months after the launch of an ORS and zinc promotion campaign in Nepal, use of ORS with zinc rose from virtually zero to 12%.^14^ In Benin, zinc use in project districts rose from 32% to 54%, and ORS use rose from 40% to 58%, following a combination of interventions over 2 years. These interventions included providing zinc to public- and private-sector facilities, pharmacies, and outlets; training public- and private-sector providers; and conducting a mass media campaign.^15^

This study evaluates the effect of a comprehensive USAID-funded program that introduced zinc and promoted its use with ORS in Ghana beginning in late 2011. The Strengthening Health Outcomes through the Private Sector (SHOPS) program in Ghana was implemented in close collaboration with the Ghanaian Ministry of Health and other regulatory agencies such as the Pharmacy Council and the Food and Drugs Authority. Programmatic lessons learned from this evaluation may inform the implementation of childhood diarrhea treatment programs in other settings.

## PROGRAM DESCRIPTION

### Ghana Context

Diarrheal diseases are the fourth leading cause of child mortality in Ghana, accounting for an estimated 9% of all mortality among children under 5 years of age.^16^ In the 2014 Ghana Demographic and Health Survey (DHS), prevalence of diarrhea among children under 5 in the 2 weeks preceding the survey was 12%.^17^ Our analysis of the 2014 DHS data showed that 43% of caregivers who seek diarrhea treatment for their child do so in the private sector, while 53% seek care in the public sector and the remaining 4% go to other sources such as traditional practitioners. The same analysis showed that private pharmacies and over-the-counter medicine sellers (OTCMS)—the primary drug-dispensing outlets at the community level—are by far the most commonly used private sources for diarrhea treatment accessed by caregivers of children under 5 years. Together, they account for over half of visits to private-sector providers for childhood diarrhea.

Diarrheal diseases are the fourth leading cause of child mortality in Ghana.

In 2010, Ghana’s government adopted the WHO/UNICEF guidelines that recommend treating acute diarrhea in children under 5 with a new, lower-osmolarity ORS plus 20 mg of zinc supplements (10 mg for children younger than 6 months). By 2011, the Ghana Health Service (the service delivery arm of the Ghana Ministry of Health) had created the policies and protocols needed to adopt the recommendations outlined in the joint WHO/UNICEF statement on diarrhea management and, with UNICEF funding, was prepared to train public-sector staff and procure dispersible zinc tablets for public-sector facilities. In addition, the Ghana Food and Drugs Authority had approved local production of dispersible zinc tablets (which prior to September 2011 were unavailable in public-sector facilities or in the private sector) and low-osmolarity ORS. These actions were critical in creating an enabling environment for the successful launch of a private-sector zinc program in Ghana.^18^

Ghana adopted the WHO/UNICEF diarrhea treatment guidelines in 2010.

### The SHOPS Project in Ghana

Between September 2011 and September 2015, the USAID-funded SHOPS project collaborated with the Ghanaian Ministry of Health and other local partners to develop and implement a comprehensive program for introducing zinc and the new diarrhea treatment guidelines to the private sector as a complement to public-sector efforts. The SHOPS project included (1) working with local pharmaceutical manufacturers to ensure supply and availability of quality, affordable zinc, (2) training private health care providers and providing supportive supervision to improve their childhood diarrhea management practices, and (3) developing and airing a targeted national mass media campaign to generate demand for zinc and promote its use alongside ORS to treat childhood diarrhea. Most of the SHOPS activities were national in scope; however, at the request of USAID, SHOPS focused provider training and supportive supervision in USAID’s 3 priority regions (Greater Accra, Western, and Central), which account for approximately one-third of the country’s population.^19^ SHOPS focused its provider efforts primarily on OTCMS, as they are the most commonly used private-sector source for diarrhea treatment.

### Building a Viable Zinc Market and Ensuring Supply and Availability

SHOPS partnered with local pharmaceutical manufacturer M&G Pharmaceuticals Ltd. (M&G) to encourage its entry into the commercial market and to help create demand for its zinc product, Zintab. SHOPS worked with M&G to develop a marketing plan and an innovative strategy to distribute zinc products nationally through commercial channels, particularly into rural areas. SHOPS worked primarily with commercial wholesalers. In addition, it facilitated a distribution partnership between M&G and local NGOs in hard-to-reach rural areas. The NGOs, who were in the business of distributing health commodities, procured ORS and zinc from M&G and resold them to local OTCMS. In January 2012, M&G entered the private commercial market, beginning distribution in certain areas of the country. By the end of 2013, M&G was also supplying its zinc products to public-sector health facilities. In 2014, zinc was available in the public and private sectors in USAID’s 3 priority regions, and by the end of the SHOPS project in 2015, zinc was available nationwide.

The SHOPS project partnered with a local pharmaceutical manufacturer in Ghana to create demand for its zinc product.

### Provider Training and Supportive Supervision

Zinc programs worldwide have faced challenges in changing provider behaviors with regard to diarrhea management. In USAID’s 3 priority regions of Ghana, the SHOPS project collaborated with the Ghana Health Service, the Ghana Pharmacy Council, professional associations, and other stakeholders to implement an innovative partnership approach to improve providers’ diarrhea management practices. Between 2012 and 2014, this approach included:

- Developing standard training curricula for nonclinical personnel working at the community level (such as OTCMS, pharmacy technicians, and community health workers) as well as for clinical providers.
- Training frontline private-sector providers (OTCMS and pharmacists) in the priority regions during March and April 2012. M&G made Zintab available for sale to providers at the end of each training session, and trainees were given information on where to purchase additional supplies of zinc. SHOPS also partnered with the Pharmacy Council to offer refresher trainings to OTCMS.
- Extending the diarrhea management training to other private providers including midwives, physician assistants, pharmacy technicians, and dispensing technicians.
- Implementing a supportive supervision program aimed at Pharmacy Council inspection teams. These teams, who visit OTCMS at least twice per year, were provided with training and a mobile phone-based supportive supervision tool so they could answer questions and provide on-the-job training to OTCMS in diarrhea management.
- Implementing a mobile phone text message campaign that provided systematic reminders, via interactive quiz questions, to OTCMS during diarrhea season to reinforce key messages from the trainings.

**Figure:**
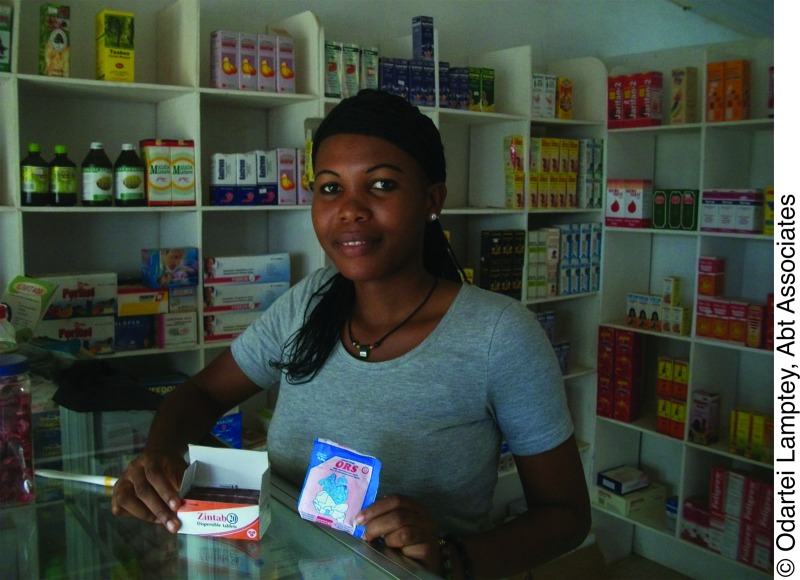
An over-the-counter medicine seller in Ghana sells oral rehydration salts (ORS) and Zintab, a zinc product manufactured locally by M&G Pharmaceuticals.

### Mass Media Campaign and Other Demand-Generation Activities

SHOPS partnered with the USAID-funded Ghana Behavior Change Support (BCS) project to conduct a national mass media campaign, featuring television and radio advertisements designed to increase awareness among both caregivers and service providers of the new diarrhea treatment protocols for children under 5. SHOPS and BCS developed the content of the campaign materials, and a local advertising agency designed and pretested them. Launched in July 2012, the mass media campaign ran annually during the diarrhea season, from April to October. The campaign provided information on the effectiveness of ORS and zinc for treating diarrhea and how to correctly administer both products. Job aids, treatment guideline wall charts, and client brochures created through this partnership were distributed widely for use in pharmacies and all OTCMS shops, and by M&G’s sales teams.

**Figure:**
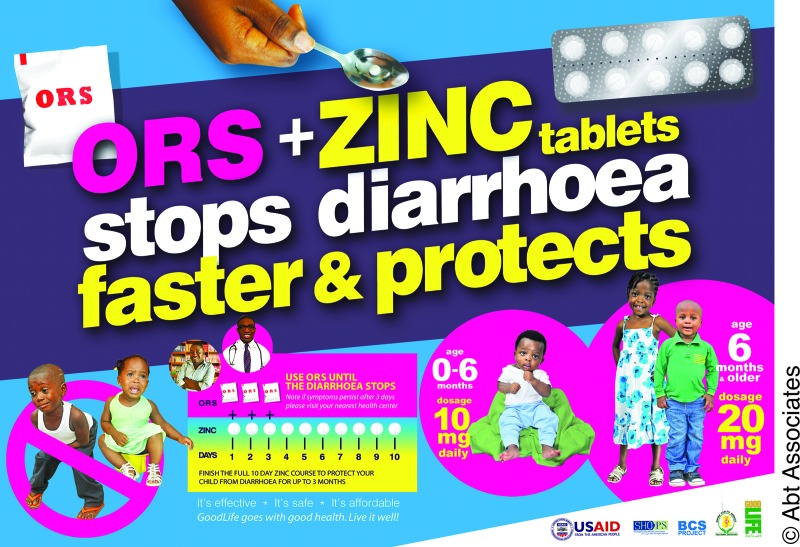
A poster advertising oral rehydration salts (ORS) and zinc, part of the SHOPS mass media campaign in Ghana, was distributed widely to pharmacies and over-the-counter medicine sellers to improve caregivers’ and providers’ awareness of the new diarrhea treatment protocols for children under 5.

### Other Zinc Promotion Programs in Ghana

To the best of our knowledge, the SHOPS interventions were the *only* comprehensive and ongoing ORS and zinc promotion activities taking place in the 3 target regions during the study period, and the only activities working with the private sector. Procurement processes for the purchase of zinc for public-sector use were initiated during 2011–2012, and zinc was thereafter made available at public-sector facilities nationally. In addition, during 2012, with UNICEF and USAID funding, the Ghana Health Service revised training curricula on childhood diarrhea management and then conducted a one-time training on diarrhea management with ORS and zinc targeting public-sector pediatricians, general practitioners, midwives, and community health nurses from all 10 regions.

## METHODS

### Study Design and Procedures

We used a pre-post study design to examine changes in diarrhea treatment practices among caregivers over time. We administered 2 cross-sectional household surveys of caregivers: a baseline survey at the beginning of SHOPS interventions (May–June 2012), and a follow-up survey just over 2 years later (August–September 2014).

We administered both surveys in the 3 USAID target regions (Greater Accra, Western, and Central) during Ghana’s rainy seasons (April–July and September–November), when diarrhea prevalence is highest. Eligible survey respondents were caregivers of children ages 6–59 months who reported that their child had had an episode of diarrhea (defined as having 3 or more loose or watery stools over the course of 1 day) in the previous 2 weeks. We excluded children under the age of 6 months because the prevalence of acute diarrhea is lower in this age group than in the 6–59-month age group.

We contracted with a data collection firm based in Accra to train data collectors, pilot test the instruments, and conduct the fieldwork. All interviews were done face-to-face in local languages (Twi, Fante, and Ga) and/or English.

### Sampling

#### Sample Size and Power Calculations

We developed sample size estimates for the household surveys based on practical considerations, including budget and time constraints. Accordingly, we determined a sample size of 750 caregivers for each survey across all 3 study regions. We calculated the minimum detectable effect to ensure that the sample was large enough to show changes in key indicators between baseline and follow-up. With a sample of 750 caregivers in each survey, the minimum detectable effect with 80% power was estimated to be a 2.2 percentage point increase in zinc use.

#### Sampling Design

We used a multi-stage sampling approach to select the sample of caregivers for each survey. In Ghana, regions are divided into districts, which are in turn divided into enumeration areas (EAs). At each stage (region, district, EA, household), we first selected the number of sampling units and then allocated the target sample of 750 caregivers among these units according to the following procedure:

**Stage 1:** **Regional strata.** We divided the 3 study regions into 4 sampling strata: Accra metropolitan, Accra nonmetropolitan, Central, and Western. For each survey, we allocated the target sample of 750 caregivers to each stratum in proportion to its population, using population census data.^19^

**Stage 2:** **Districts.** We selected a sample of 15 districts from the 44 total districts in the 4 strata—using the same districts in the baseline and follow-up surveys. We included the Accra Metropolitan district as the only district in its stratum. The remaining 14 districts were selected using stratified probability proportional to size (PPS) sampling, where size was the population of the district. For each district, we allocated the target sample of caregivers in proportion to the population of that district.

**Stage 3: Enumeration areas.** We divided each of the 15 selected districts into urban and rural EAs (where applicable), selecting a total of 4 EAs in each district (2 urban and 2 rural) using equal probability sampling. (If a district had only urban or rural EAs, we selected 4 urban or 4 rural EAs.) This yielded a total of 60 EAs across all 15 districts. We divided the target sample of caregivers allocated to each district equally among the 4 EAs selected for that district. We supplemented the list of 60 EAs with a randomly selected list of additional urban and rural EAs, to be assigned to districts where the allocated sample size of eligible caregivers could not be met in implementing the survey. In the end, 70 EAs were selected for the baseline survey and 84 EAs were selected for the follow-up survey.

**Stage 4: Households.** Using detailed EA maps and a landmark (school, church, mosque, etc.) as a starting point, data collection teams went door-to-door to screen every household and identify those with at least 1 caregiver of a child aged 6–59 months who experienced diarrhea in the prior 2 weeks. As soon as an eligible caregiver was identified, the interviewers administered the survey. Screening and surveying in each EA stopped once the target sample of surveyed eligible caregivers was met; supplemental EAs were used if the target could not be met in a given EA.

**Stage 5: Caregivers.** If an eligible household contained more than 1 caregiver of a child aged 6–59 months who experienced diarrhea in the prior 2 weeks, the interviewers randomly selected 1 of the caregivers for the survey using a Kish grid.^20^ Similarly, if the selected caregiver had more than 1 child aged 6–59 months with diarrhea in the prior 2 weeks, the interviewers used a Kish grid to randomly select only 1 child for the survey.

**Final samples.** Our final sample sizes were 754 caregivers in the baseline survey and 751 in the follow-up survey. Refusal rates were less than 0.5% in each survey. The household sampling was done independently for each survey; however, it is possible that some of the same households participated in both the baseline and follow-up surveys given that we visited the same areas.

### Survey Instruments

We developed 3 study instruments: (1) a household listing and screening form; (2) a baseline survey instrument; and (3) a follow-up survey instrument. Data collectors used the household listing and screening form to screen households in each EA and to determine their eligibility for the study. To ensure comparability of baseline and follow-up data, the baseline and follow-up survey instruments were nearly identical, with a few additional questions included in the follow-up survey. To minimize misclassification of treatments and misreporting, the interviewers used visual aids containing photos of popular drugs and treatments currently on the market in Ghana. Whenever possible, the interviewer asked the caregiver to show the treatment packaging to confirm which treatments were used.

### Variables

Our main outcome variables of interest were the proportion of caregivers reporting use of ORS, zinc, ORS with zinc, antibiotics, and antidiarrheals at each point in time.

At both baseline and follow-up, we also collected demographic and socioeconomic data (caregiver’s sex, age, education level, and marital status, and child’s age), diarrhea characteristics (duration in days, presence of fever, presence of blood), sources of treatment, and data on household assets. We constructed a wealth index by adapting a methodology applied in El-Khoury et al.^21^ and Pitchforth et al.^22^ (See supplementary material for more information.) For each household in the sample, we recorded the asset variables collected in the surveys, then assigned a population-level wealth quintile to each caregiver in the sample. In the follow-up survey, we asked respondents about their recall of diarrhea and zinc messages in the past month.

### Analytic Methods

We first conducted chi-square and *t* tests to determine whether our baseline and follow-up samples were balanced in terms of certain observable demographic and socioeconomic characteristics.

We then conducted bivariate and multivariate regression analyses using Stata software to estimate changes in caregiver treatment behaviors between baseline and follow-up.^23^ We ran ordinary least squares (OLS) regressions on the pooled sample of caregivers from the baseline and follow-up surveys (N = 1,505), using the following model:

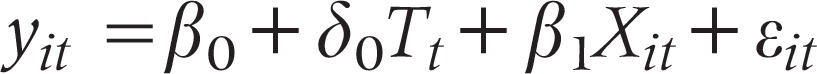


Where:

- y_it_ is the binary dependent variable (outcome) for caregiver *i* at time *t* (where *t* indicates either baseline or follow-up).
- T_t_ is a binary variable equal to 1 for the follow-up period and 0 for the baseline.
- X_it_ is a vector of covariates for caregiver *i* at time *t*.

Covariates included caregiver characteristics (sex, age, education, marital status), child age, diarrhea characteristics (duration in days, presence of fever, presence of blood), household characteristics (wealth index score, wealth quintile), and district of residence (in order to adjust for potential district effects).

Then:

- β_0_ is the conditional average value of the outcome at baseline.
- (β_0_ + δ_0_) is the conditional average value of the outcome at follow-up.
- δ_0_ is the estimate of interest, which measures the conditional difference in the value of the outcome between baseline and follow-up.

We ran the model on the 5 main outcome variables mentioned above. For each outcome variable, we ran the regression with and without the vector of covariates (X).

Antibiotics are generally considered inappropriate to use in cases of non-bloody diarrhea. We analyzed changes in antibiotic use over time among caregivers who did *not* report blood with their child’s diarrhea episode and tested whether outcomes differed among this subgroup. We implemented this analysis by interacting T_t_ with a dummy variable indicating the presence of blood in our model and testing for significance of the interaction term on antibiotic use. In addition, we analyzed the group of caregivers who used ORS with zinc in combination with antibiotics at follow-up, according to whether the diarrhea episode was accompanied by blood in stool and/or fever.

Finally, we used a chi-square test to examine whether recall of zinc messages was associated with zinc use at follow-up.

All variables were weighted using sampling weights that conformed to our sampling strategy.

### Ethical Approval

The study was reviewed by Abt Associates’ Institutional Review Board and was granted exemption. The study was also reviewed and approved by the Ghana Health Service Ethical Review Committee. SHOPS obtained oral informed consent from respondents, and those who did not provide consent were not surveyed.

## RESULTS

### Characteristics of Study Sample

The baseline and follow-up samples were balanced with respect to most observable characteristics (Table 1). In both samples, about 98% of caregivers were female, with an average age of just over 31 years. A higher percentage of caregivers in the follow-up sample were married than in the baseline sample (84% vs. 77%, respectively; *P* = .07). The majority of caregivers in both samples had completed at least primary school.

**TABLE 1:** Characteristics of Study Samples in Ghana at Baseline (2012) and Follow-Up (2014)

Characteristics	Baseline (N = 754)	Follow-Up (N = 751)	*P* Value
**Caregiver**			
Female, %	97.9	97.8	.98^a^
Age, mean (SD), years	31.1 (9.1)	31.5 (9.1)	.64^a^
Married, %	77.0	84.0	.07*^a^
Education, % distribution			.16^b^
None	15.4	15.3	
Primary	26.2	20.7	
Completed primary or some middle	43.9	43.5	
Completed middle or some secondary	13.9	18.5	
Completed secondary or above	0.6	2.1	
**Child**			
Child age, mean (SD), months	29.3 (15.4)	28.0 (13.9)	.31^a^
Diarrhea duration, mean (SD), days	4.5 (3.0)	4.3 (3.3)	.64^a^
Diarrhea with fever, %	43.8	43.5	.95^a^
Diarrhea with blood, %	11.4	5.6	.095*^a^
**Household**			
Wealth index score,^c^ mean (SD)	0.58 (0.2)	0.59 (0.1)	.70^a^
Wealth quintile, % distribution			.008***^b^
First (poorest)	23.4	14.3	
Second	19.9	32.6	
Third	42.1	46.2	
Fourth	14.1	10.3	
Fifth (wealthiest)	0.6	0.3	

Abbreviation: SD, standard deviation.

*** *P*< .01,

** *P*< .05

* *P*< .10.

a *P* value from *t* test.

b *P* value from chi-square test.

c Wealth index score ranges from 0 to 1.

There was no difference in mean duration of the diarrhea episode between baseline and follow-up (4.5 and 4.3 days, respectively) or in the proportion of children who had fevers during the diarrhea episode (43.8% and 43.5%, respectively). However, approximately twice as many children in the baseline sample had blood in their stool compared with those in the follow-up sample (11.4% vs. 5.6%, respectively; *P* = .095). Since presence of blood in the stool typically warrants antibiotic treatment, caregivers in the baseline sample should have been more likely to use antibiotics over other treatments compared with caregivers in the follow-up sample.

While the constructed *wealth index* was the same, on average, across both samples (score of 0.58 out of 1 in the baseline sample and 0.59 in the follow-up sample), there was a statistically significant difference in the *distribution of wealth* between baseline and follow-up (*P* = .008). The follow-up sample was more likely to include caregivers from the second population wealth quintile and less likely to include caregivers from the first (poorest) wealth quintile. These differences in the wealth distribution may have affected treatment behavior across the 2 samples, especially if costs of treatments were prohibitive for those belonging to the poorest quintile.

We included all variables in Table 1, including presence of blood in the stool and wealth quintiles, as covariates in the multivariate regression analysis.

### Changes in Diarrhea Management Practices Among Caregivers

Table 2 shows changes in caregiver diarrhea treatment practices between baseline and follow-up. Each row shows the results of a separate regression run on each outcome of interest, without covariates (Panel A) and with covariates (Panel B).

**TABLE 2:** Changes in Caregiver Diarrhea Management Practices in Ghana Between Baseline (2012) and Follow-Up (2014) (N = 1,505)

	(A) Bivariate Regression Results				(B) Multivariate Regression Results^a^		
Treatment	Baseline (β_0_)	Follow-Up (β_0_ + δ_0_)	Difference Over Time (δ_0_)	*P* Value	Follow-Up (β_0_ + δ_0_)	Difference Over Time (δ_0_)	*P* Value
(1) Zinc (with or without ORS)	0.013	0.313	0.300***	<.001	0.321	0.308***	<.001
(2) ORS (with or without zinc)	0.377	0.599	0.222***	<.001	0.611	0.234***	<.001
(3) ORS with zinc	0.008	0.292	0.284***	<.001	0.301	0.293***	<.001
(4) Antibiotics	0.662	0.382	-0.280***	<.001	0.350	-0.312***	<.001
(5) Antidiarrheals	0.102	0.051	-0.051**	.02	0.074	-0.028	.13

Abbreviation: ORS, oral rehydration salts.

*** *P*< .01,

** *P*< .05,

* *P*< .10.

a Covariates included in multivariate regressions are caregiver characteristics (sex, age, education, marital status), child age, diarrhea characteristics (duration in days, presence of fever, presence of blood), household characteristics (wealth index score, wealth quintile), and district fixed effects.

Results showed a large, positive, and statistically significant increase in use of recommended diarrhea treatments between baseline and follow-up, both with and without covariates. The bivariate regression results show that use of zinc rose from 1.3% at baseline to 31.3% at follow-up—an increase of 30.0 percentage points (*P*<.001) (Panel A, Row 1). Use of ORS rose from 37.7% to 59.9%—an increase of 22.2 percentage points (*P*<.001) (Panel A, Row 2). Importantly, use of the recommended combination treatment, ORS with zinc, rose 28.4 percentage points (*P*<.001), from 0.8% at baseline to 29.2% at follow-up (Panel A, Row 3). About 93% of caregivers who used zinc at follow-up used it in combination with ORS, as recommended. The size and statistical significance of these results remained largely the same after including covariates to adjust for possible confounding factors (Panel B). We found that the wealth index and wealth quintiles were not significant contributors to zinc and ORS use.

Use of ORS and zinc increased substantially and significantly between baseline and follow-up.

Results also showed significant declines in caregiver use of antibiotics and antidiarrheals. The bivariate regression results show that antibiotic use dropped by 28 percentage points (*P*<.001), from 66.2% at baseline to 38.2% at follow-up (Panel A, Row 4). When adding covariates, the reduction in antibiotic use was even higher, at 31.2 percentage points (*P*<.001) (Panel B, Row 4). Antidiarrheal use decreased by 5.1 percentage points (*P*<.05), from 10.2% at baseline to 5.1% at follow-up (Panel A, Row 5). When adding covariates, the decline was around half as great (2.8 percentage points) and was not statistically significant (Panel B, Row 5).

Use of antibiotics declined significantly between baseline and follow-up.

We found no evidence that trends in antibiotic use were different among the subgroup of caregivers who did not report blood with the diarrhea episode. The coefficient on the interaction term was not statistically significant (*P* = .49). The reduction in antibiotic use among caregivers who did not report blood in the stool was estimated at 31.9 percentage points (*P*<.001) (not shown).

In some cases, caregivers reported using antibiotics in addition to ORS and zinc. Overall, in the follow-up survey, 21.8% of caregivers who used ORS and zinc also used antibiotics. Table 3 analyzes the group of caregivers who used ORS with zinc *in combination with antibiotics* at follow-up (n = 52), according to whether the diarrhea episode was accompanied by blood in stool and/or fever. Close to half of this group (43.2%) reported that their child had neither a fever nor blood in the stool with the episode of diarrhea, suggesting that antibiotics should not have been used.

43% of caregivers who used ORS with zinc in combination with antibiotics said their child did not have blood in the stool or a fever, suggesting inappropriate antibiotic use.

**TABLE 3:** Characteristics of Diarrhea Episode Among Caregivers in Ghana Who Gave ORS With Zinc in Combination With Antibiotics at Follow-Up (2014) (N = 52)

Characteristics of Diarrhea Episode	Value
Diarrhea with no blood in stool or fever, %	43.2
Diarrhea with fever only, %	49.0
Diarrhea with blood in stool only, %	3.0
Diarrhea with blood in stool and fever, %	4.7
Diarrhea duration, mean (SD), days	4.3 (2.5)

Abbreviations: ORS, oral rehydration salts; SD, standard deviation.

### Sources of Diarrhea Treatment

At follow-up, about 64% of caregivers sought treatment from the private sector and 36% sought treatment from the public sector. Specifically, 54% of caregivers who reported using ORS with zinc at follow-up sought treatment from the private sector (mainly private pharmacies or OTCMS), while 46% sought treatment from the public sector. About 61% of caregivers who used antibiotics at follow-up sought treatment from the private sector and 39% from the public sector.

### Recall of Zinc Messages and Use of Zinc

At follow-up, we examined whether respondents recalled having heard any messages about using zinc for diarrhea treatment. About 36% of caregivers at follow-up had heard or seen at least 1 zinc message, primarily from television and radio. Caregivers who recalled hearing or seeing any zinc message in the last month were more than 3 times as likely to use zinc (55%) as those who did not recall hearing zinc messages (18%) (*P* = .003). We recognize that this correlation is not indicative of causality.

## DISCUSSION

This evaluation revealed that diarrhea treatment behaviors in the 3 study regions in Ghana improved substantially in the 3 years following the implementation of a comprehensive ORS and zinc promotion program. The program included building a local zinc market, training private providers on zinc and ORS, and implementing a mass media campaign. Caregivers reported significantly higher levels of use of ORS with zinc and lower levels of antibiotic use, even after adjusting for potential confounding factors. Previous studies in Benin, Bangladesh, and Nepal found comparable effect sizes, ranging from 5.0 to 22.5 percentage point increases in caregiver use of zinc.^12^^–^^14^ Our findings are an important contribution to the limited evidence base on the effectiveness of ORS and zinc promotion interventions.

To the best of our knowledge, this study is one of very few evaluations of a comprehensive program seeking to improve diarrhea management. While the study was not designed to evaluate the relative effectiveness of each component of the program, our results suggest that a similar package of interventions has the potential to be applied in other settings where rapid scale-up of use of ORS with zinc is desired.

Our pre-post study design is unable to determine how much of the observed increase in ORS and zinc use is solely attributed to the SHOPS program. We adjusted for possible confounding factors in the multivariate regression analysis; however, there may be other unobservable variables that affected results, including possible spillover effects from other diarrhea management interventions in other regions of Ghana. It is possible, for instance, that the 2012 USAID and UNICEF-funded public-sector trainings, mentioned earlier in this article, may have contributed to increased awareness and use of correct diarrhea treatments in the country. We posit, however, that the SHOPS interventions were likely important drivers of the changes observed in the 3 study regions. This is primarily because the SHOPS interventions were the only ongoing and comprehensive ORS and zinc promotion activities taking place in the survey regions during the study’s time frame. Indeed, the public-sector trainings in 2012 were not followed-up with any additional interventions during the study period.

Finally, even though SHOPS primarily focused the provider training component of its interventions on the private sector, caregivers obtained zinc and ORS from both private- and public-sector providers. Thus, engaging both the private and public sectors will be essential to ensure increased access to ORS and zinc.

Because caregivers obtained zinc and ORS from both private and public providers, engaging both sectors will be essential to ensure increased access to and use of appropriate diarrhea treatment.

Recall of zinc messages from the mass media campaign was positively associated with caregiver use of zinc to treat diarrhea. Similar associations were observed in both Nepal^14^ and Benin.^15^ Due to the cross-sectional nature of our surveys, we cannot assess the directionality of this association: does using zinc predispose a caregiver to remember zinc messages, or does recalling a zinc message predispose a caregiver to use (or report having used) zinc? There may also be other factors, not controlled for in this analysis, that are associated with both zinc use and recall of media messages and that could affect the likelihood of seeking treatment. Further study is required to determine whether this is a causal link and to identify the specific messages and channels that resonate most with different groups of caregivers.

While antibiotic use decreased substantially from baseline to follow-up, it remained high, even among the subgroup of caregivers who did not report blood with the diarrhea episode. In addition, a high proportion of caregivers who gave ORS with zinc reported treating with antibiotics as well, even in the absence of blood or fever symptoms. It is important to note that our evaluation was not designed to comprehensively assess correct antibiotic use. For instance, some children presenting with both diarrhea and fever may have had another infection (such as otitis media or pneumonia) that warranted antibiotic prescription but was not specifically asked about in our survey. Still, high antibiotic use is a persistent problem in many countries. In Benin, for example, while zinc use grew from 32% to 54% from 2009 to 2011, the proportion of zinc users who also used an antibiotic grew from 11% to 39% during the same time period.^15^ In Bihar, India, while the percentage of zinc use tripled from 2011 to 2013, the percentage of antibiotic use also doubled in the same time period—although this increase might reflect better classification of diarrhea treatments (i.e., fewer unknowns) in the 2013 survey.^11^ A recent qualitative study in Ghana found that caregivers were accustomed to using antibiotics and felt strongly about continuing to use them.^24^ On the other hand, OTCMS’ lack of knowledge of the reasons for limiting antibiotics use and their inadequate negotiation skills also played a big role in the incorrect provision of antibiotics.^24^ The SHOPS trainings for private OTCMS emphasized that antibiotics should not be prescribed for acute diarrhea without blood in the stool and should only be used when appropriate, i.e., in the presence of bloody diarrhea or shigellosis, per WHO/UNICEF guidelines.^2^ The trainings also highlighted problems associated with inappropriate use of antibiotics, and SHOPS reinforced these messages during post-training supportive supervision visits. Yet, through mystery client surveys conducted in 2014, the project found that 29% of trained providers (data not published) continue to incorrectly prescribe antibiotics. Additional targeted studies, from both the caregiver and provider (prescribing) perspectives, are needed to refine the estimates of levels of incorrect antibiotic use, to better understand reasons for this ongoing area of concern, and to develop interventions to address these issues.

While antibiotic use decreased substantially, it remained high at follow-up.

### Study Limitations

It is worth noting a few important limitations to our study. First, the pre-post design precludes attributing observed changes in outcomes to the interventions. While we controlled for possible confounding factors in the regression analysis, other unobservable factors may have influenced the results. Second, the baseline and follow-up surveys were conducted during different seasons (May–June and August–September). It is possible that some seasonal factors, such as nutritional status, disease incidence, and household disposable income, may have differed between the 2 seasons and therefore have affected the results. Third, all outcome variables are based on recall of survey respondents and may therefore be subject to recall bias and information bias due to the misclassification of certain treatments. We attempted to mitigate these risks by using an elaborate visual aid to assist caregivers in accurately identifying treatments and by building verification questions into the survey instrument.

## CONCLUSION

From 2011 to 2015, the USAID-funded SHOPS project implemented a comprehensive program in Ghana to introduce and promote the use of ORS and zinc to treat acute childhood diarrhea. The SHOPS package of interventions included the development and airing of a mass media campaign to generate demand for ORS and zinc as well as working with private-sector providers, public-sector policy makers, and local manufacturers to increase availability of affordable zinc to caregivers of children under 5. This study showed substantial increases in the use of ORS and zinc by caregivers and decreases in antibiotics use. A similar package of interventions has the potential to be applied in other settings to achieve rapid scale-up of use of ORS and zinc. Additional global efforts need to be dedicated to further reduce persistent use of antibiotics, especially in cases of non-bloody diarrhea. Although the SHOPS project has ended, USAID is currently supporting another program to scale up use of ORS and zinc in Ghana, and 2 local manufacturers, M&G and Phyto-Riker, are manufacturing and distributing zinc to the private and public sectors. Going forward, an important area of research would be to determine which of the program components are most cost-effective and potentially most scalable, to help donors and program implementers prioritize investments amid shrinking budgets.

## Supplementary Material

supplementary material

## Notes

**Peer Reviewed**
