# Supplementary material for: Improved Childhood Diarrhea Treatment Practices in Ghana: A Pre-Post Evaluation of a Comprehensive Private-Sector Program
Source: Glob Health Sci Pract. 2016 Jun 20;4(2):264–75. doi: 10.9745/GHSP-D-16-00021 (PMC4982250; doi:10.9745/GHSP-D-16-00021)
Supplement: supplementary material [file GHSP-D-16-00021_index.html]

Supplement to Improved Childhood Diarrhea Treatment Practices in Ghana: A Pre-Post Evaluation of a Comprehensive Private-Sector Program | Global Health: Science and Practice

## GHSP-D-16-00021 Supplementary Material

Sloane et al. doi: 10.9745/GHSP-D-16-00021

- Supplementary Material - Sloane et al. doi: 10.9745/GHSP-D-16-00021
