# Supplementary material for: Improved Childhood Diarrhea Treatment Practices in Ghana: A Pre-Post Evaluation of a Comprehensive Private-Sector Program
Source: Glob Health Sci Pract. 2016 Jun 20;4(2):264–75. doi: 10.9745/GHSP-D-16-00021 (PMC4982250; doi:10.9745/GHSP-D-16-00021)
Supplement: supplementary material [file 16-00021-Sloane-Supplementary-Material.pdf]

El-Khoury M, Banke K, Sloane P. Improved childhood diarrhea treatment practices in Ghana: a pre-post evaluation of a comprehensive private-sector program. Glob Health Sci Pract. 2016;4(2).
http://dx.doi.org/10.9745/GHSP-D-16-00021

SUPPLEMENTARY MATERIAL. Construction of a Wealth Index for the Ghana Childhood Diarrhea Management Intervention

El-Khoury et al.[1] and Pitchforth et al.[2] calculate a wealth index using a subset of the household asset indicators used in constructing the Demographic and Health Survey (DHS) wealth index; they then rank households in the sample according to population-level wealth quintiles. Using a similar approach, we gathered data on the same household asset indicators collected in the 2008 Ghana DHS.[3] Using the 2008 Ghana DHS data set, we ran a regression of the DHS wealth index on the household asset indicators. The fitted values from that regression constituted our "proxy" wealth index (R^2 value of 0.94). Next, we used the proxy wealth index to rank women in the DHS sample, group them into quintiles, and identify cutoff values of the proxy index for each quintile. We then used the estimated coefficients from the DHS regression described above as weights to construct a comparable wealth index in our caregiver baseline and follow-up samples, and rescaled the index to vary between 0 and 1. We used the cutoff values identified in the DHS sample described above to classify our caregiver survey respondents into population wealth quintiles, where quintile 1 represents the lowest wealth quintile and quintile 5 represents the highest wealth quintile in our sample.

REFERENCES

1. El-Khoury M, Hatt L, Gandaho T. User fee exemptions and equity in access to caesarean sections: an analysis of patient survey data in Mali. Int J Equity Health. 2012;11(1):49. CrossRef. Medline
2. Pitchforth E, van Teijlingen E, Graham W, Fitzmaurice A. Development of a proxy wealth index for women utilizing emergency obstetric care in Bangladesh. Health Policy Plan. 2007;22(5):311-319. Medline
3. Ghana Statistical Service; Ghana Health Service; ICF International. Ghana demographic and health survey, 2008. Rockville (MD): ICF International; 2009. Available from: http://www.dhsprogram.com/pubs/pdf/FR221/FR221[13Aug2012].pdf
